# Splenic B1 B Cells Acquire a Proliferative and Anti-Inflamatory Profile During Pregnancy in Mice

**DOI:** 10.3389/fimmu.2022.873493

**Published:** 2022-04-28

**Authors:** Natalin J. Valeff, María S. Ventimiglia, Marcos Dibo, Udo R. Markert, Federico Jensen

**Affiliations:** ^1^ Laboratorio de Inmunología de la Reproducción, CEFYBO-UBA-CONICET, Buenos Aires, Argentina; ^2^ Placenta Lab, Department of Obstetrics, University Hospital Jena, Jena, Germany; ^3^ Centro Integrativo de Biología Y Química Aplicada, Universidad Bernardo O’Higgins, Santiago, Chile

**Keywords:** B cells, B1 B cells, pregnancy, autoimmunity, tolerance

## Abstract

B cells are a heterogeneous cell population with differential ontogeny, anatomical location, and functions. B1 B cells are a distinct subpopulation characterized by their unique capacity of self-renewal, the production of large quantities of IL-10, and the ability to secrete protective, anti-inflammatory natural antibodies (NAbs), presumably upon down-regulation of CD1d expression. Although natural antibodies are thought to be protective, due to their polyreactivity, their participation in certain autoimmune diseases has been suggested. In the context of pregnancy, the role of B1 B cells has been discussed controversially. While in human pregnancies B1 B cells and natural/polyreactive antibodies they produce are involved in the development of preeclampsia, in mice they promote healthy gestation and fetal protection. In this work, we aimed to functionally characterize the splenic B1 B cell population during pregnancy in mice. Functional enrichment analysis using only up-regulated transcripts from a transcriptomic profile performed on total splenic B cells from pregnant compared to non-pregnant mice showed augmented cell cycle and DNA replication pathways. Proliferation studies by flow cytometry showed augmented Ki-67 proliferation marker expression and percentages of B1 B cells. Furthermore, B1 B cells produced higher levels of IL-10 and lower levels of TNF-α leading to an increased IL-10/TNF-α ratio and showing an immunoregulatory phenotype. Finally, we observed lower expression of CD1d on B1 B cells, suggesting a higher capacity to produce NAbs in the context of pregnancy. In summary, our results showed not only an expanded and proliferative splenic B1 B cell population during pregnancy but also the acquisition of immunomodulatory capacities suggesting its critical role in the intricate process of pregnancy tolerance.

## Introduction

As essential components of the adaptive immune system, B cells are mostly recognized by their role as mediators of humoral immune responses through antibody production (1). However, upon activation, B cells can also produce a wide range of cytokines (2) as well as present antigens to T cells, thus playing a critical role in orchestrating an immune response (3–5).

B cells represent a heterogeneous cell population that based on their ontogeny, anatomical location and cellular markers can be divided into three different subsets: B1 B cells, follicular B cells (FO), and marginal zone (MZ) B cells (6, 7). Particularly, B1 B cells originate mainly during embryonic development from precursors present in the yolk sac and fetal liver, and their numbers are maintained by cell division in adulthood (6, 8, 9). Although B1 B cells are mainly located in peritoneal and pleural cavities (10, 11), in mice (12), they can also be found in the spleen and can be distinguished from B2 B cells by their low expression of B220 (9, 13, 14). One of the main features of the B1 B cell population is the production of natural antibodies (NAbs), which are produced in the absence of antigenic stimulus (15). Albeit the molecular mechanism regulating the production of NAbs by B1 B cells is not fully understood, it appears to be associated with a down-regulation of CD1d (16). NAbs participate in many physiological processes, including regulation of homeostasis, prevention of inflammation, and infections (17). In addition, due to their polyreactivity, NAbs and B1 B cells are involved in autoimmunity (18). Besides, upon activation, B1 B cells produce large quantities of IL-10, a potent anti-inflammatory cytokine (19–22). In the context of pregnancy, the role of B1 B cells has been evaluated controversially. Indeed, it was proposed that B1 B cells can produce natural/polyreactive antibodies which may be involved in the onset of preeclampsia (23), a pregnancy-associated disorder with suspected autoimmune origin (24). On the other hand, B1 B cells were shown to control the differentiation of pro-inflammatory Th1 and Th17 cells, thus promoting a healthy gestation in mice (25). Furthermore, IL-10 producing B1 B cells protect the fetus from rejection after adoptive transfer into an abortion-prone mice model (26).

In the work presented here, we aimed to identify and functionally characterize the splenic B1 B cell population in pregnant mice.

## Results

### Splenic B1 B Cells Acquire a Proliferative State During Pregnancy

**Figure 1:**
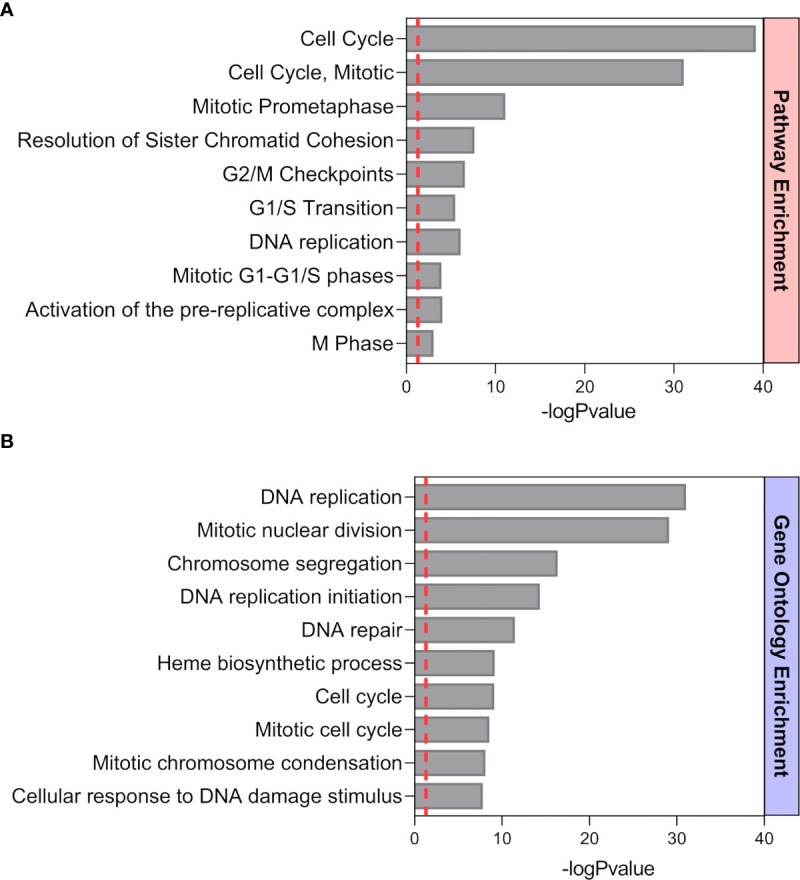
Functional Pathway **(A)** and GO **(B)** enrichment analysis of up-regulated genes from a previously performed transcriptomic profiling (24). Pathways and biological processes up-regulated in splenic B cells from P mice (n=4) compared to NP (n=4) animals. The ten most significant pathways and biological processes that showed a positive modulation are sorted by statistical significance using the -log_10_ p-value (BH p< 0.05, fold changes > 1.5).

**Figure 2:**
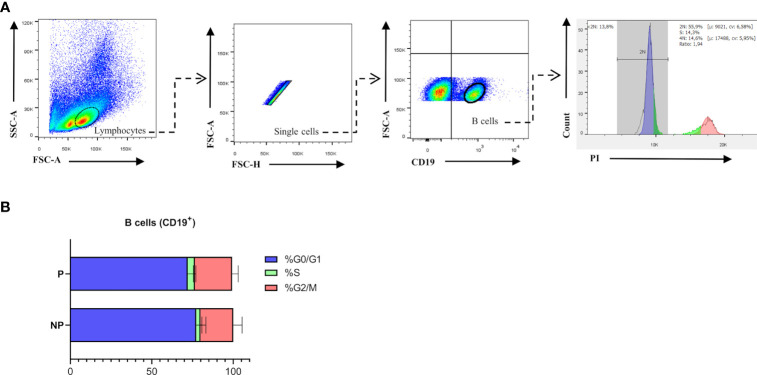
**(A)**. Cell Cycle gating strategy for B cell analysis by flow cytometry. Representative pseudocolor and contour plots showing gating strategy used to quantify percentages of cell cycle phases in B cells from spleens of pregnant (n=5) and non-pregnant (n=5) mice. Lymphocytes were gated by forward scatter (FSC) vs. side scatter (SCC), doublets were eliminated and CD19 was used to define the total B cell population in the spleen. Over gated B cells (CD19^+^), a representative histogram of propidium iodide (PI) staining was used to evaluate phase G0/G1 in (blue), phase S (green), and phase G2/M (red). Auto-fluorescence of each biological replicate was used as negative control for CD19. **(B)**. Comparison of B cell cycle analysis in pregnant and non-pregnant mice. Bar plots show a tendency of arrest in the S and G2 phase of B cells (CD19^+^) from pregnant (P) (G0/G: 72.8±6; S: 4.4±0.5; G2/M: 22.8±3.7; n=5) compared to non-pregnant (NP) (G0/G:77.3±3.5; S:2.6±0.9; G2/M:20±3.1; n=5) mice. Two-way ANOVA (α=0.05).

**Figure 3:**
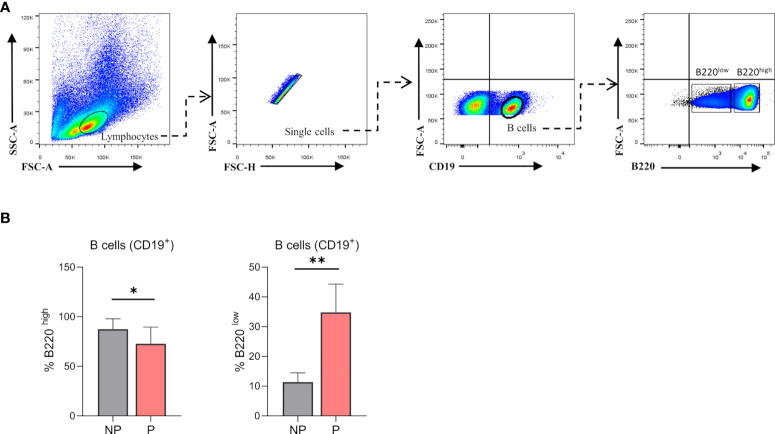
**(A)**. Gating strategy. Representative contour plots showing gating strategy used to quantify percentages of B1 and B2 B cells in the spleen of pregnant (P) and non-pregnant (NP) mice. Lymphocytes were gated by FSC vs. SCC, doublets were eliminated and CD19 was used to define the total B cell population in the spleen. In the B cell (CD19^+^) gate, B220 was used to determine B1 (CD19^+^B220^low^) and B2 (CD19^+^B220^high^) B cell populations. Auto-fluorescence of each biological replicate was used as a negative control for CD19 and B220. **(B)**. Comparison of percentages of splenic B1 and B2 B cells in pregnant and non-pregnant mice. Splenic B1 B cells (CD19^+^B220^low^) were significantly increased in P (34.8±4.3; n=5) as compared to NP (11.35±1.6; n=4) mice. Splenic B2 B cells (CD19^+^B220^high^) were significantly diminished in P (72.8±6.4; n=7) as compared to NP (87.5±3.9; n=7) mice. Unpaired Student’s t-test (α=0.05), (*) p<0.05, (**) p<0.01.

**Figure 4:**
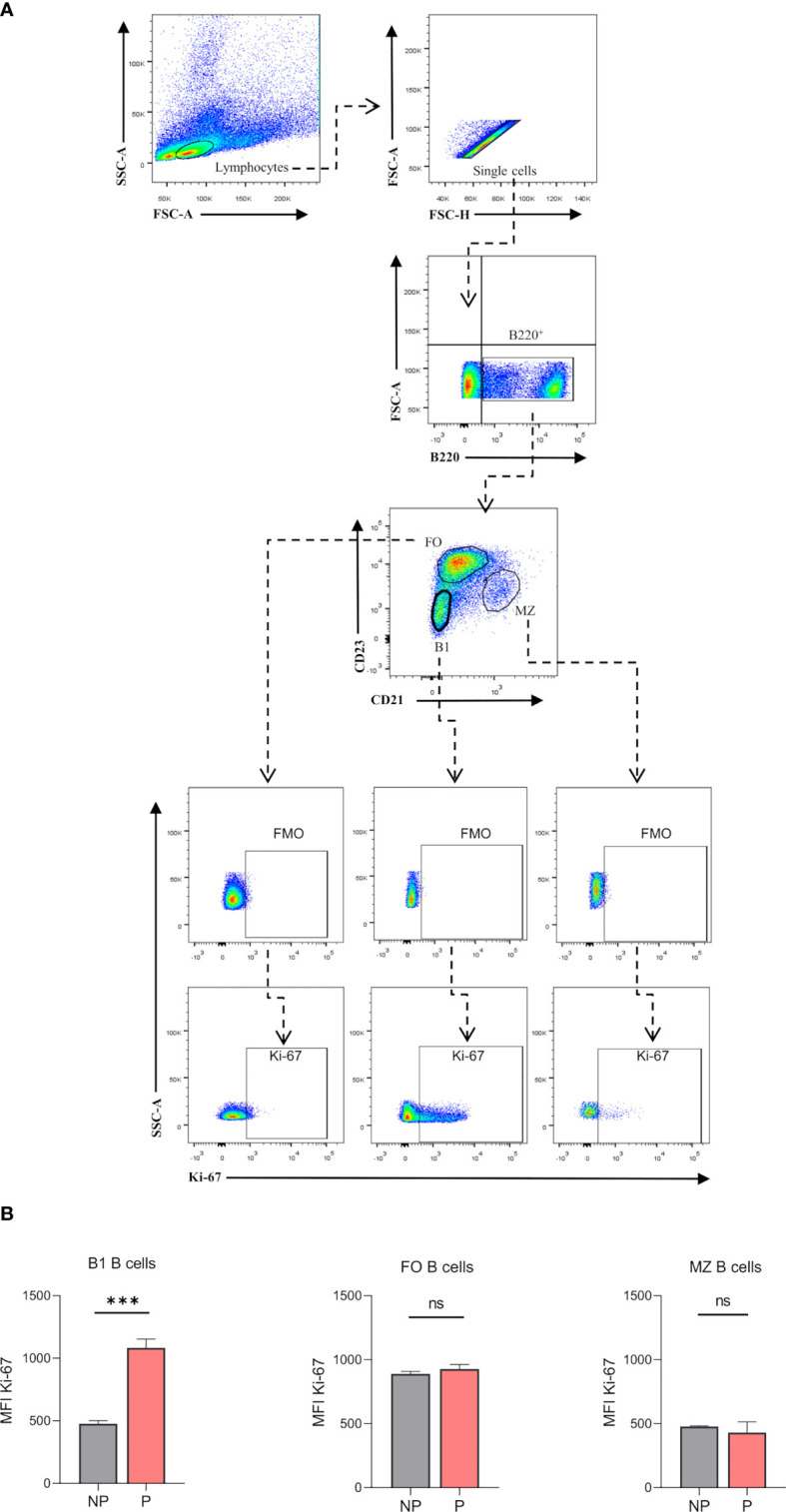
**(A)**. Gating strategy to evaluate proliferation marker Ki-67 on splenic B cell subsets. Representative pseudocolor plots showing gating strategy used to quantify Ki-67 expression of B cell subsets in the spleen of pregnant (P) and non-pregnant (NP) mice. Lymphocytes were gated by FSC vs. SCC, doublets were eliminated and B220 was used to define the total B cell population in the spleen. Auto-fluorescence of each biological replicate was used as a negative control for B220. Additionally, Ki-67 expression was analyzed in B1 (B220^+^CD23^-^CD21^-^), FO (B220^+^CD23^+^CD21^-^), and MZ (B220^+^CD23^-^CD21^high^) B cells, using fluoresce minus one (FMO) as control. Back gating of B1 B cells showed 95% of cells with low expression of B220 (B220^low^CD23^-^CD21^-^), while B2 B cells showed high expression levels of B220 marker (data not shown). **(B)**. Analysis of Ki-67 expression on B cell subsets. Ki-67 (MFI) on splenic B1 B cells was significantly increased in P (1083±41.2; n=3) as compared to NP (476.3±14,6; n=3) mice. Splenic B2 B cells showed no significant differences in Ki-67 MFI from P compared to NP mice. Unpaired Student’s t-test (α=0.05), ns= no significant, (***) p<0.001.

Previously published enrichment analysis of differentially expressed genes (DEGs) from transcriptomic profiling performed on total isolated splenic B cells showed augmented cell cycle and DNA replication pathways from pregnant (P) as compared to NP mice (27). Here, further functional enrichment analyses using only up-regulated transcripts in total isolated B cells from pregnant (P) compared to non-pregnant mice (NP) were performed. Pathway enrichment showed over-represented pathways related to B cell proliferation, involved in the cell cycle (REACTOME: 17635), mitotic cell cycle (REACTOME: 16819), G2/M cell cycle checkpoints (REACTOME: 18178), and DNA replication (REACTOME: 17107) (**Figure 1A**). Furthermore, Gene Ontology enrichment showed over-represented biological processes in B cells isolated from pregnant (P) compared to non-pregnant mice (NP): mitotic nuclear division (GO:0007067), chromosome segregation (GO:0007059), and DNA replication initiation (GO:0006270) (**Figures 1A, B**). The 10 most significantly over-represented biological processes and biological pathways are listed in **Supplementary Tables 1**, **2**. To understand the biological meaning of these modifications, we next compared cell cycle phases in total CD19^+^ B cells (**Figure 2A**) from the spleen of P and NP mice. Although a tendency of arrest at G2/M and S phase in P mice was observed, this difference did not reach statistical significance (**Figure 2B**). Additionally, flow cytometry analysis showed two distinct populations of CD19^+^ B cells detected by their differential expression of the marker B220 (**Figure 3A**). Percentages of CD19^+^B220^high^ B cells were significantly decreased (p<0.05) while percentages of CD19^+^B220^low^ B cells, whose phenotype corresponds to that of B1 cells (9, 13, 14), were significantly increased (p<0.01) in P mice (**Figure 3B**). Given these changes, the expression level of the proliferation marker Ki-67 was evaluated on splenic B cell subpopulations (**Figure 4A**). To do so, we used different cellular markers that permit the discrimination between B1 and B2 (FO and MZ) B cells. Flow cytometry analysis of B cell subsets showed increased Ki-67 expression in B220^low^CD23^-^CD21^-^ B1 B cells from P (p<0.001) as compared to NP mice (**Figure 4B**). No differences were observed in terms of Ki-67 expression in FO (B220^high^CD23^+^CD21^-^) and MZ (B220^high^CD23^-^CD21^high^) B cells from P and NP mice (**Figure 4B**).

### Splenic B1 B Cells Showed an Anti-Inflammatory Profile During Pregnancy

**Figure 5:**
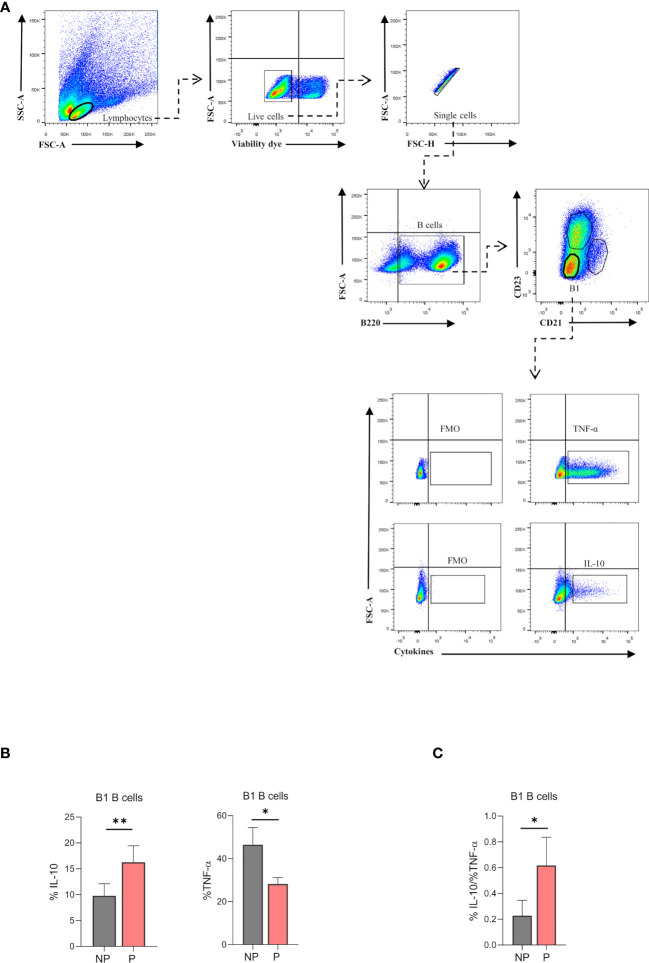
**(A)**. Gating strategy to evaluate intracellular cytokine production in splenic B1 B cells. Representative pseudocolor plots showing the gating strategy used to evaluate IL-10 and TNF-α producing-B1 B cells from the spleen of pregnant (P) and non-pregnant mice (NP). Lymphocytes were gated by FSC vs. SCC, and dead cells and doublets were eliminated. Then B220 CD23, and CD21 markers were used to define the B1 B cell subset (B220^low^CD23^-^CD21^-^). Auto-fluorescence of each biological replicate was used as a negative control for B220, CD23, and CD21. Additionally, IL-10 and TNF-α production was assessed in B1 B cells (B220^low^CD23^-^CD21^-^) using FMO as control. **(B)**. Percentages of B1-IL-10 and B1-TNF-α^+^ B cells in pregnant and non-pregnant mice. B1-IL10 B cell (B220^low^CD23^-^CD21^-^IL-10^+^) percentage was significantly increased in pregnant (P) (16.2±1.4; n=5) as compared to non-pregnant (NP) (9.8 ± 1.04; n=5) mice. Additionally, B1-TNF-α B cell (B220^low^CD23^-^CD21^-^TNF-α^+^) percentage was significantly diminished in P (28.2±3; n=5) as compared to NP (46.5 ± 8; n=5) mice. **(C)**. Ratio of B1-IL-10^+^ and B1-TNF-α^+^ B cells in pregnant and non-pregnant mice. The ratio of B1-IL10/ B1-TNF-α B cells was significantly increased in P (0.61±0.1; n=5) as compared to NP (0.23 ±0.06; n=5) mice. Unpaired Student’s t-test (α=0.05), (*) p<0.05, (**) p<0.01.

To evaluate the immune profile of the expanded B220^low^CD23^-^CD21^-^ splenic B1 B cell population during pregnancy, intracellular production of IL-10 and TNF-α was measured by flow cytometry upon LPS stimulation *in vitro*. Percentages of B220^low^CD23^-^CD21^–^–IL-10 producing B1 B cells were significantly increased (p<0.01) in P compared to NP mice (**Figure 5A**). In addition, percentages of B220^low^CD23^-^CD21^–^–TNF-α producing B1 B cells were significantly decreased (p<0.05) in P as compared to NP mice (**Figure 5B**). More importantly, the ratio between IL-10 and TNF-α, which indicates the inflammatory profile of the cells, showed an increased anti-inflammatory capacity of B1 B cells during pregnancy (**Figure 5C**).

### Splenic B1 B Cells Lower CD1d Expression During Pregnancy

**Figure 6:**
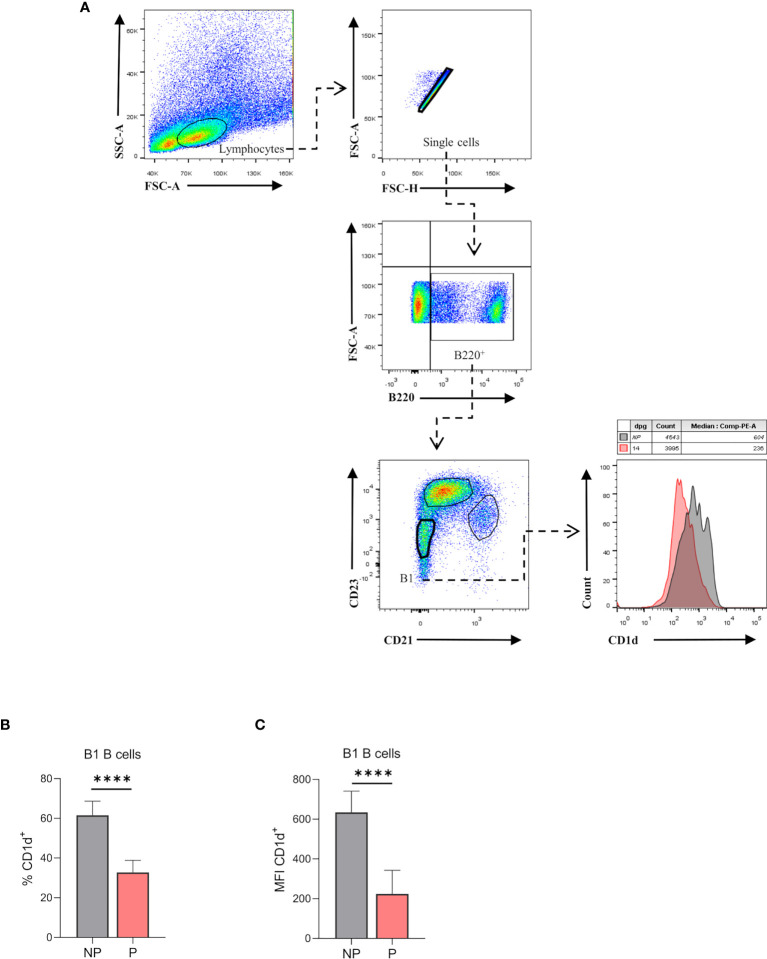
**(A)**. Percentages of CD1d expressing-B1 B cells in pregnant and non-pregnant mice. Representative pseudocolor plots showing gating strategy used to quantify CD1d expression on B1 B cells from the spleen of pregnant (P) and non-pregnant mice (NP). Lymphocytes were gated by FSC vs. SCC, and dead cells and doublets were eliminated. Then B220 CD23, and CD21 markers were used to define the B1 B cell subset (B220^low^CD23^-^CD21^-^). Auto-fluorescence of each biological replicate was used as a negative control for B220, CD23, and CD21. Additionally, CD1d expression was assessed in this population, using FMO as control. **(B)**. Percentages of B1-CD1d B cells in pregnant and non-pregnant mice. Percentages of B1-CD1d B cells were significantly diminished in pregnant (P) (37.8 ± 2.4; n=5) as compared to non-pregnant (NP) (61.6 ±3.1; n=5) mice. **(C)**. Mean fluorescence intensity (MFI) of CD1d on B1 B cells in pregnant and non-pregnant mice. MFI of CD1d on B1 B cells (B220^low^CD23^-^CD21^-^) was significantly diminished in P (224±42; n=5) as compared to NP (634.4±47.7; n=5) mice. Unpaired Student’s t-test (α=0.05), (****) p<0.0001.

Recently, it has been demonstrated that the production of natural antibodies by B1 B cells is associated with a reduction of their CD1d expression (16). Hence, we evaluated the expression of CD1d on B220^low^CD23^-^CD21^-^ splenic B1 B cells from P and NP mice by flow cytometry (**Figure 6A**). As shown in **Figure 6B**, percentages of B220^low^CD23^-^CD21^-^CD1d^+^ were significantly lower (p<0.0001) in P mice as compared to NP mice (**Figure 6B**). Similarly, CD1d expression (MFI) on B220^low^CD23^-^CD21^-^ B1 B cells was also significantly lower in P mice than in NP control animals (**Figure 6C**).

## Discussion

In this study, we have demonstrated that during pregnancy in mice, splenic B1 B cells are expanded and acquire an anti-inflammatory profile.

Pregnancy in mammals represents a unique challenge for the maternal immune system as it should be able to tolerate the developing semi-allogenic fetus and prevent rejection reactions. This requires temporal modifications in the innate as well as the adaptive immunity (28). Among others, maternal B cell depletion has been proposed as a potential mechanism to avoid immune self-reactivity which could compromise pregnancy well-being (29). However, in contrast, the expansion of certain B cell populations, namely regulatory B cells and MZ B cells, has been previously described in pregnant mice. This expansion has been suggested to be part of the intricate mechanisms of immune tolerance induced during gestation (23, 25, 30). Similarly, here we have shown an *in-silico* analysis of a transcriptomic profile established in pure isolated total splenic B cells from P and NP mice, showing an up-regulation of genes and pathways associated with cell cycle and DNA replication in B cells during gestation. Interestingly, when we analyzed expression levels of the proliferation marker Ki-67 in main B cell populations in the spleen, only B1 B cells, but not FO or MZ B cells, showed an increased expression of Ki-67 in P mice compared to NP animals, suggesting that only B1 B cells are boosted to proliferate during gestation. Indeed, percentages of B1 B cells were significantly higher in the spleen of P mice as compared to NP animals. From a functional point of view, B-1 B cells were shown to regulate immunity by their special cytokine secretion pattern (15). B1 B cells constitutively produce IL-10, which seems to switch them towards regulatory functions (15, 31). Furthermore, B1 B cell-derived IL-10 production has been associated with attenuated responses to infections with *Leishmania* (32, 33) and atherosclerosis (34) and seems to enhance B1 B cell expansion *via* induction of proliferation (35). Here we have demonstrated that B1 B cells from P mice produced significantly higher levels of IL-10 compared to B1 B cells from NP animals, suggesting that during pregnancy, B1 B cells boost their immune regulatory capacity. In addition, the higher production of IL-10 by B1 B cells from P mice could also provide a mechanistic explanation for the higher proliferation rate as well as the expanded proportion of B1 B cells observed during pregnancy. The potent anti-inflammatory cytokine IL-10 is a critical component in the process of pregnancy tolerance as it regulates the inflammation elicited by paternal antigen recognition (36). Indeed, serum levels of IL-10 were shown to be significantly increased in women coursing normal pregnancies but not in those pregnancies destined for loss (37). On the other hand, the pro-inflammatory cytokine TNF-α undergoes the opposite kinetics, expressed at lower levels in women coursing normal pregnancies and at higher levels in those suffering pregnancy loss (37). Moreover, the IL-10/TNF-α ratio is increased in normal pregnancies but not in women with pregnancy failure (37). In this line, the present study did not only demonstrate a higher IL-10 and lower TNF-α expression in B1 B cells during pregnancy, but also an increased IL-10/TNF-α ratio, thus, positioning the B1 B cells as central components for the cytokine balance required for pregnancy wellbeing.

A major function of B1 B cells is the generation of natural antibodies (NAbs), mainly IgM and IgG_3_, which are produced in the absence of antigen stimulation (15). The exact mechanisms that induce NAbs production remain incompletely understood. However, recently, it has been demonstrated that the suppression of CD1d expression on splenic B1 B cells is associated with NAbs production (16). In the present study, we found a significant reduction in the expression of CD1d in B1 B cells during normal pregnancy, suggesting an enhanced capacity of NAbs production during gestation. Supporting this idea, we have previously demonstrated that serum levels of both, IgM and IgG_3_, are significantly higher in pregnant women compared to non-pregnant controls (38). Similarly, using a mouse model of pregnancy disorders, we also have demonstrated that serum levels of IgM are significantly higher in normal pregnant mice compared to non-pregnant and pregnant mice suffering pregnancy failures (39). It is worth mentioning that B1 B cells and natural/auto-antibodies they produce have previously been implicated in the onset of preeclampsia (PE), a pregnancy-associated syndrome with a strong inflammatory profile and suspected autoimmune origin (23). Nevertheless, in the mentioned work, the numbers, as well as the functionality of B1 B cells, were measured in diagnosed PE patients, opening the question of whether they are involved in the dysregulated mechanisms that trigger the onset of the disease or if its consequence. Further studies are demanded to better understand the role of B1 B cells in PE patients.

Although the presented experiments have been performed in mice and several substantial molecular differences between human and murine pregnancies have been reported (40), we expect transferability of our observations to humans.

Overall, data presented here position the B1 B cells as key players in cytokine balance required for the immunological equilibrium in pregnancy. Further studies are demanded to better understand the biological significance of B1 B cell expansion as well as NAbs production during pregnancy.

## Material and Methods

### Animals

Seven to eight weeks old virgin C57BL/7 females were allogeneically mated 1:1 with BALB/c males until pregnancy was confirmed (detection of vaginal plug). The day of vaginal plug detection was considered as day 0 of pregnancy (P). In all experiments, pregnant animals were sacrificed on day 14 of pregnancy, the same gestational day we previously performed gene-array analysis on total CD19^+^ splenic B cells. Non-pregnant virgin age-matched C57BL/7 females were used as controls (NP). All mice were maintained in the facilities of the Center of Pharmaceutical and Botanical Studies (CEFYBO) of the Faculty of Medicine, Buenos Aires University under a 12-hour light/12-hour dark cycle and were given *ad libitum* access to food and water. Animal experiments were carried out according to institutional guidelines after ministerial approval (CICUAL resolution number 2248).

### GO and PA Enrichment Analysis

For biological interpretation of the DEGs (27), significantly over-represented gene ontology (GO) terms and biological pathways (PA) were explored with the InnateDB analysis tool. Over-representation analysis was performed using a hypergeometric algorithm with the Benjamini-Hochberg (BH) multiple test correction (1.5<fold-change cutoff <-1.5 and expression p-value cutoff P < 0.05). GO and PA were considered significantly over-represented with an FDR <0.05 and a -log p-value threshold >1.3.

### Antibodies and Reagents

The following fluorescence labeled antibodies were purchased from BioLegend, San Diego, CA 92121, USA: anti-mouse/human B220 (clone RA3-6B2), anti-mouse CD19 (clone 6D5), anti-mouse CD23 (clone B3B4), anti-mouse CD21/35 (clone 7E9), anti-mouse CD1d (clone 1B1), anti-mouse Ki-67 (clone 16A8), anti-mouse TNF-a (clone MP6-XT22), anti-mouse IL-10 (clone JES5-16E3), Zombie Violet viability dye (423113).

Additionally, propidium iodide (PI, Sigma Aldrich, Saint Louis, MO 63103, USA), RPMI 1640 medium (Gibco, Life Technologies, NY, US), fetal bovine serum (FBS, Internegocios, BA, Argentina), penicillin/streptomycin (Gibco, Life Technologies, NY, US), phorbol 12-myristate 13-acetate (PMA, Sigma Aldrich, Saint Louis, MO 63103, USA), ionomycin (Sigma Aldrich, Saint Louis, MO 63103, USA), lipopolysaccharide (LPS, E. coli 055:B5, Sigma Aldrich, Saint Louis, MO 63103, USA) and brefeldin A (BFA, BioLegend, San Diego, CA 92121, USA) were used to perform the next experiments.

### Cell Preparation and Flow Cytometry

#### Cell Cycle With PI

For cell cycle analysis, 1x10^6^ total splenocytes from P and NP were incubated for 30 min at 4 °C with anti-CD19 antibodies. After washing, cells were fixed with ethanol solution (EtOH 70% in cold), drop to drop over vortex to avoid clumping, and stored at 4°C in the dark. On the day of the FACS analysis, cells were incubated for 30 min at 37°C with propidium iodide (PI) solution (50 µg/ml). After several washes, the cellular pellet was resuspended in FACS buffer and stored at 4°C. Data were acquired on a FACS Canto II (BD Biosciences) and the mean fluorescence intensity (MFI) of PI in total B cells was analyzed by using FlowJo v10 software.

#### Ki-67 Expression

For Ki-67 expression analysis on B cell subsets, 1x10^6^ splenocytes from P and NP were stained 30 min at 4 °C with anti-B220, anti-CD23, and anti-CD21 antibodies to identify FO, MZ, and B1 B cell populations. After washing, cells were fixed with ethanol solution (EtOH 70% in cold), drop to drop over vortex to avoid clumping, and stored at -20°C 1h in the dark. After washing, cells were stained for 30min with a Ki-67 antibody solution at room temperature in the dark. After several washes, the cellular pellet was resuspended in FACS buffer and stored at 4°C. Fluorescence minus one (FMO) was used as a control. Data were acquired on a FACS Canto II (BD Biosciences) and the mean fluorescence intensity of ki-67 in B cell populations was analyzed by using FlowJo v10 software.

#### Percentages of B Cell Populations and CD1d Expression

For flow cytometry analysis, single-cell suspension was obtained from spleens of P and NP mice following previously described procedures (25, 39). Briefly, spleens were crushed in a 100 μm cell strainer to obtain a single cell suspension and red blood cells were lysed in lysis buffer (NH4Cl 1,5 M, KHCO3 0,1 M, EDTA 0,9 M) for 5 min. After washing, cell suspensions were counted using a Neubauer chamber, and 1×10^6^ cells were stained for 30 min at 4 °C with labeled specific antibodies. After several washings, the cellular pellet was resuspended in FACS buffer and stored at 4°C. Data were acquired on a FACS Canto II (BD Biosciences) and analyzed by using FlowJo v10 software.

#### Cell Culture and Intracellular Cytokine Production

For intracellular cytokine analysis, 1x10^6^ splenocytes from P and NP were cultured *in vitro* in a 24 well plate in 1 ml RPMI 1640 medium supplemented with FBS (fetal bovine serum, 10%) and penicillin/streptomycin (1%) and stimulated with lipopolysaccharide (LPS; 10µg/ml) for 24 h. During the last 5 h of culture, phorbol 12-myristate 13-acetate (PMA, 50 ng/ml), ionomycin (1µg/ml), and brefeldin A (BFA, 1 µg/ml) were added. Cells were collected and incubated with Zombie Violet viability dye for 30 min at room temperature in the dark. After several washes, cells were stained with a solution of specific antibodies (anti-B220, anti-CD23, anti-CD21) for 30 min at 4°C, and fixed with PFA (1%, ON, 4°C). After washing and permeabilization (saponin 0,1%, 10 min, 4°C, dark), cells were intracellularly stained with anti-IL-10 and anti-TNF-α fluorescence-labeled antibodies. After several washes, the cell pellet was resuspended in FACS buffer and stored at 4°C. Fluorescence minus one (FMO) was used as a control. Data were acquired on FACS Canto II (BD Biosciences) and analyzed by using FlowJo v10 software.

### Statistical Analysis

Data were analyzed with PRISM software (ver. 8.0; GraphPad). Normality was checked using D’Agostino & Pearson normality test, or the Shapiro-Wilk normality test. Unpaired t-test (normally distributed data) or Mann–Whitney U-test (non-normally distributed data) were applied to compare two groups, p < 0.05 was considered statistically significant. Analysis of variance (ANOVA) followed by Tukey multiple tests was applied as appropriate to evaluate differences of means of multiple groups. Significant differences between groups are indicated with asterisks (*P<0.05; **P<0.01; ***P< 0.001, ****P<0.0001).

## Data Availability Statement

Publicly available datasets were analyzed in this study. This data can be found here: https://www.ncbi.nlm.nih.gov/geo/query/acc.cgi?acc=GSE174290.

## Ethics Statement

The animal study was reviewed and approved by CICUAL, Medical Faculty, Buenos Aires University (CICUAL resolution number: 2248).

## Author Contributions

NV designed experiments, performed experiments, analyzed data, and wrote the manuscript. MSV collaborated in some experiments and helped to draft the manuscript; MD analyzed data and helped to draft the manuscript. UM analyzed data and critically reviewed and discussed the prefinal version of the manuscript. FJ designed experiments, analyzed data, contributed with reagents, supervised the work, and wrote the manuscript. All authors contributed to the article and approved the submitted version.

## Funding

This study was supported by grants from Agencia Nacional de Promoción Científica y Tecnológica PICT (2016/004), PICT (2016-201-0151), and PICT-PRH (2016-004) to FJ.

## Conflict of Interest

The authors declare that the research was conducted in the absence of any commercial or financial relationships that could be construed as a potential conflict of interest.

## Publisher’s Note

All claims expressed in this article are solely those of the authors and do not necessarily represent those of their affiliated organizations, or those of the publisher, the editors and the reviewers. Any product that may be evaluated in this article, or claim that may be made by its manufacturer, is not guaranteed or endorsed by the publisher.
