# Supplementary material for: Splenic B1 B Cells Acquire a Proliferative and Anti-Inflamatory Profile During Pregnancy in Mice
Source: Front Immunol. 2022 Apr 28;13:873493. doi: 10.3389/fimmu.2022.873493 (PMC9095819; doi:10.3389/fimmu.2022.873493)
Supplement: Supplementary file 1 [file Table_1.docx]

| **Pathway Name** | **Pathway Id** | **-log10 (p)** | **Source Name** | **Gene Symbols** |
| --- | --- | --- | --- | --- |
| Cell Cycle | 17635 | 39,2 | REACTOME | Alms1; Aurka; Birc5; Blm; Bora; Brca1; Brca2; Bub1b; Casc5; Ccna2; Ccnb1; Ccnb2; Ccne1; Ccne2; Cdc20; Cdc25c; Cdc45; Cdc6; Cdca5; Cdca8; Cdk1; Cenpa; Cenph; Cenpi; Cenpk; Cenpl; Cenpm; Cenpn; Cenpp; Cep290; Cep70; Cep76; Chek1; Chek2; Cks1b; Clspn; Dbf4; Dhfr; Dna2; Esco2; Fbxo5; Gins1; Gmnn; H2afz; Hist1h2ak; Hist1h2bb; Hist1h2bh; Hist1h2bm; Hist2h2bb; Hist3h2ba; Incenp; Kif18a; Kif20a; Kif23; Kif2c; Kntc1; Mad2l1; Mastl; Mcm10; Mcm2; Mcm3; Mcm4; Mcm6; Mcm7; Mcm8; Mis18bp1; Mlf1ip; Mybl2; Ncapd2; Ncapg2; Ncaph; Nek2; Nuf2; Nup37; Oip5; Orc1; Orc2; Pcna; Plk1; Plk4; Pola1; Pole; Pole2; Ppp2r1b; Prim1; Prkar2b; Rad51; Rfc4; Rpa3; Rrm2; Sgol1; Sgol2; Ska1; Smc2; Spc24; Spc25; Syce2; Tfdp1; Top2a; Tyms; Ube2c; Wee1; Zwilch; Zwint; |
| Cell Cycle, Mitotic | 16819 | 31,1 | REACTOME | Alms1; Aurka; Birc5; Bora; Bub1b; Casc5; Ccna2; Ccnb1; Ccnb2; Ccne1; Ccne2; Cdc20; Cdc25c; Cdc45; Cdc6; Cdca5; Cdca8; Cdk1; Cenpa; Cenph; Cenpi; Cenpk; Cenpl; Cenpm; Cenpn; Cenpp; Cep290; Cep70; Cep76; Cks1b; Dbf4; Dhfr; Dna2; Esco2; Fbxo5; Gins1; Gmnn; H2afz; Hist1h2ak; Incenp; Kif18a; Kif20a; Kif23; Kif2c; Kntc1; Mad2l1; Mastl; Mcm10; Mcm2; Mcm3; Mcm4; Mcm6; Mcm7; Mcm8; Mlf1ip; Mybl2; Ncapd2; Ncapg2; Ncaph; Nek2; Nuf2; Nup37; Orc1; Orc2; Pcna; Plk1; Plk4; Pola1; Pole; Pole2; Ppp2r1b; Prim1; Prkar2b; Rfc4; Rpa3; Rrm2; Sgol1; Sgol2; Ska1; Smc2; Spc24; Spc25; Tfdp1; Top2a; Tyms; Ube2c; Wee1; Zwilch; Zwint; |
| Mitotic Prometaphase | 18604 | 11,1 | REACTOME | Birc5; Bub1b; Casc5; Ccnb1; Ccnb2; Cdc20; Cdca5; Cdca8; Cdk1; Cenpa; Cenph; Cenpi; Cenpk; Cenpl; Cenpm; Cenpn; Cenpp; Incenp; Kif18a; Kif2c; Kntc1; Mad2l1; Mlf1ip; Ncapd2; Ncaph; Nuf2; Nup37; Plk1; Ppp2r1b; Sgol1; Sgol2; Ska1; Smc2; Spc24; Spc25; Zwilch; Zwint; |
| Resolution of Sister Chromatid Cohesion | 17085 | 7,63 | REACTOME | Birc5; Bub1b; Casc5; Ccnb1; Ccnb2; Cdc20; Cdca5; Cdca8; Cdk1; Cenpa; Cenph; Cenpi; Cenpk; Cenpl; Cenpm; Cenpn; Cenpp; Incenp; Kif18a; Kif2c; Kntc1; Mad2l1; Mlf1ip; Nuf2; Nup37; Plk1; Ppp2r1b; Sgol1; Sgol2; Ska1; Spc24; Spc25; Zwilch; Zwint; |
| G2/M Checkpoints | 18178 | 6,56 | REACTOME | Ccnb1; Ccnb2; Cdc25c; Cdc45; Cdc6; Cdk1; Chek1; Chek2; Clspn; Dbf4; Mcm10; Mcm2; Mcm3; Mcm4; Mcm6; Mcm7; Mcm8; Orc1; Orc2; Rfc4; Rpa3; Wee1; |
| DNA Replication | 17107 | 6,09 | REACTOME | Ccna2; Cdc45; Cdc6; Dbf4; Dna2; Gins1; Gmnn; Mcm10; Mcm2; Mcm3; Mcm4; Mcm6; Mcm7; Mcm8; Orc1; Orc2; Pcna; Pola1; Pole; Pole2; Prim1; Rfc4; Rpa3; |
| G1/S Transition | 18725 | 5,49 | REACTOME | Ccna2; Ccnb1; Ccne1; Ccne2; Cdc45; Cdc6; Cdk1; Cks1b; Dbf4; Dhfr; Fbxo5; Mcm10; Mcm2; Mcm3; Mcm4; Mcm6; Mcm7; Mcm8; Orc1; Orc2; Pcna; Pola1; Pole; Pole2; Ppp2r1b; Prim1; Rpa3; Rrm2; Tfdp1; Tyms; Wee1; |
| Mitotic G1-G1/S phases | 18886 | 4,03 | REACTOME | Ccna2; Ccnb1; Ccne1; Ccne2; Cdc45; Cdc6; Cdk1; Cks1b; Dbf4; Dhfr; Fbxo5; Mcm10; Mcm2; Mcm3; Mcm4; Mcm6; Mcm7; Mcm8; Mybl2; Orc1; Orc2; Pcna; Pola1; Pole; Pole2; Ppp2r1b; Prim1; Rpa3; Rrm2; Tfdp1; Top2a; Tyms; Wee1; |
| Activation of the pre-replicative complex | 18128 | 3,93 | REACTOME | Cdc45; Cdc6; Dbf4; Mcm10; Mcm2; Mcm3; Mcm4; Mcm6; Mcm7; Mcm8; Orc1; Orc2; Pola1; Pole; Pole2; Prim1; Rpa3; |
| M Phase | 17726 | 3,06 | REACTOME | Birc5; Bub1b; Casc5; Ccnb1; Ccnb2; Cdc20; Cdca5; Cdca8; Cdk1; Cenpa; Cenph; Cenpi; Cenpk; Cenpl; Cenpm; Cenpn; Cenpp; Fbxo5; H2afz; Hist1h2ak; Incenp; Kif18a; Kif20a; Kif23; Kif2c; Kntc1; Mad2l1; Mastl; Mlf1ip; Ncapd2; Ncapg2; Ncaph; Nuf2; Nup37; Plk1; Ppp2r1b; Sgol1; Sgol2; Ska1; Smc2; Spc24; Spc25; Ube2c; Zwilch; Zwint; |

**Supplementary Table 1:** Pathway enrichment analysis

| **Gene Ontology** | **GO Id** |  | **Source** | | **Gene Symbols** | |
| --- | --- | --- | --- | --- | --- | --- |
| DNA replication | GO:0006260 | 31,0555267 | biological process | 2810417H13Rik; Blm; Brca1; Cdc6; Chaf1b; Cinp; Dbf4; Dna2; Dscc1; Dtl; Fen1; Gins1; Lig1; Mcm10; Mcm2; Mcm3; Mcm4; Mcm5; Mcm6; Mcm7; Mcm8; Nasp; Nfia; Nfix; Orc1; Orc2; Orc6; Pola1; Pole; Pole2; Polq; Rfc4; Rpa3; Rrm1; Rrm2; Ticrr; Tk1; | |  |
| Mitotic nuclear division | GO:0007067 | 29,1193844 | biological process | Anln; Aspm; Aurka; Birc5; Bora; Casc5; Ccna2; Ccnb2; Ccnf; Cdc20; Cdc25c; Cdc6; Cdca2; Cdca3; Cdca5; Cdca8; Cdk1; Cenph; Cenpn; Cenpv; Cenpw; Fbxo5; Hells; Incenp; Kif18b; Kif20b; Kif2c; Kntc1; Mastl; Mis18bp1; Ncapd2; Ncapg2; Nuf2; Nup37; Oip5; Plk1; Ran; Sgol1; Ska1; Ska3; Spc24; Spc25; Tipin; Tpx2; Wee1; Zwilch; | |  |
| Chromosome segregation | GO:0007059 | 16,3540572 | biological process | Birc5; Bub1; Casc5; Cenpf; Cenph; Cenpn; Cenpw; D2Ertd750e; Esco2; Hjurp; Incenp; Kif2c; Nek2; Nup37; Sgol1; Ska1; Ska3; Spag5; Spc25; Top2a; | |  |
| DNA replication initiation | GO:0006270 | 14,3282318 | biological process | Ccne1; Ccne2; Cdc45; Mcm2; Mcm3; Mcm4; Mcm5; Mcm6; Mcm7; Pola1; | |  |
| DNA repair | GO:0006281 | 11,4651049 | biological process | Bard1; Blm; Brca1; Brca2; Brip1; Chaf1b; Chek1; Cinp; Clspn; Eef1e1; Eme1; Exo1; Fancd2; Fanci; Fen1; Fignl1; Gen1; Kif22; Lig1; Neil3; Parpbp; Polq; Rad51; Rad51ap1; Rpa3; Rps27l; Ticrr; Uhrf1; | |  |
| Heme biosynthetic process | GO:0006783 | 9,1540179 | biological process | Alad; Alas2; Atpif1; Cpox; Fech; Slc11a2; Tmem14c; Urod; | |  |
| Cell cycle | GO:0007049 | 9,12208963 | biological process | Ccnb1; Cdc45; Chaf1b; Cinp; Cks1b; Cks2; Dbf4; Erh; Esco2; Fanci; Gmnn; Mcm2; Mcm3; Mcm4; Mcm6; Mcm7; Mcm8; Melk; Nasp; Sept8; Steap3; Tfdp1; Tfdp2; Tipin; Uhrf1; | |  |
| Mitotic cell cycle | GO:0000278 | 8,53879305 | biological process | Aurka; Ccnb1; Cdk1; Cenpf; Cenpw; Kif18b; Rrm1; Tfdp1; Tfdp2; | |  |
| Mitotic chromosome condensation | GO:0007076 | 8,10296764 | biological process | Cdca5; Ncapd2; Ncapd3; Ncapg; Ncaph; Nusap1; Smc2; | |  |
| Cellular response to DNA damage stimulus | GO:0006974 | 7,80271097 | biological process | 2810417H13Rik; 4632434I11Rik; Atad5; Blm; Brca1; Brca2; Casp3; Chek1; Chek2; Dtl; Fancd2; Mastl; Mcm7; Mcm8; Rad51; Rad54l; Rps27l; Stxbp4; Tipin; Top2a; | |  |

**Supplementary Table 2:** Gene Ontology Enrichment analysis

**Figure legends**

**Supplementary Table 1.** Pathway enrichment analysis. Detailed list of significantly over-represented biological pathways based on up-regulated DEGs in splenic B cells from P compared to NP mice sorted by p-values (BH p< 0.05, fold change > 1.5). The differentially expressed genes of each pathway are represented by gene symbols, and the significantly different biological pathways have been sorted based on the -log10 p-value.

**Supplementary Table 2.** Gene Ontology Enrichment analysis. Detailed list of significantly over-represented biological processes based on up-regulated DEGs in splenic B cells from P compared to NP mice (BH p< 0.05, fold changes (FC) > 1.5). Gene symbols define the differentially expressed genes involved in each biological process and -log p values have been used for sorting by statistical significance.
